# Green synthesis of silver nanoparticles: characterization and determination of antibacterial potency

**DOI:** 10.1007/s13204-015-0426-6

**Published:** 2015-03-20

**Authors:** Jayshree Annamalai, Thangaraju Nallamuthu

**Affiliations:** Centre of Advanced Study in Botany, School of Life Sciences, University of Madras, Guindy Campus, Chennai, 600 025 Tamil Nadu India

**Keywords:** Silver ions, Green chemistry, Human pathogens, Biomedicines

## Abstract

Silver ions (Ag^+^) and its compounds are highly toxic to microorganisms, exhibiting strong biocidal effects on many species of bacteria but have a low toxicity toward animal cells. In the present study, silver nanoparticles (SNPs) were biosynthesized using aqueous extract of *Chlorella vulgaris* as reducing agent and size of SNPs synthesized ranged between 15 and 47 nm. SNPs were characterized by UV–visible spectroscopy, scanning electron microscopy, transmission electron microscopy, X-ray diffraction and Fourier infrared spectroscopy, and analyzed for its antibacterial property against human pathogens. This approach of SNPs synthesis involving green chemistry process can be considered for the large-scale production of SNPs and in the development of biomedicines.

## Introduction

Nanotechnology is an exploitation of strange properties of materials smaller than 100 nm (nm) to create new useful objects. Nanomaterials display unique, superior and indispensable properties and have attracted much attention for their distinct characteristics that are unavailable in conventional macroscopic material. Their uniqueness arises specifically from higher surface-to-volume ratio and increased percentage of atoms at the grain boundaries. They represent an important class of materials in the development of novel devices that can be used in various physical, biological, biomedical and pharmaceutical applications.

Silver nanoparticles are nontoxic, safe inorganic antibacterial agent used for centuries and are capable of killing about 650 types of diseases causing microorganisms. Silver has been described as oligodynamic because of its ability to exert a bactericidal effect at minute concentrations. The first scientific papers describing the medical use of silver report the prevention of eye infection in neonates in 1881 and internal antisepsis in 1901 (Russell and Hugo 1994). After this, silver nitrate and silver sulfadiazine have been widely used for the treatment of superficial and deep dermal burns of wounds and for the removal of warts (Rai et al. 2009).

SNP mode of action is presumed to be dependent on Ag^+^ ions that strongly inhibit bacterial growth through suppression of respiratory enzymes and electron transport components and through interference with DNA functions (Li et al. 2006). Silver in a nanometric scale (less than 100 nm) has strong toxicity to a wide range of microorganisms (Elechiguerra et al. 2005). Morones et al. (2005) defined the antibacterial activity of SNPs in four types of Gram-negative bacteria *Escherichia coli, Vibrio cholerae, Pseudomonas aeruginosa* and *Salmonella typhi*, and suggested that SNPs attach to the surface of the cell membrane and disturb its function by penetrating into bacterial cell and release of silver ions. Silver nanoparticles have also been found to exert antibacterial activity against some drug-resistant bacteria (Birla et al. 2009; Inoue et al. 2010).

*Chlorella vulgaris* is a medicinal unicellular green microalgae, which contains more than 20 different vitamins and minerals, 19 of 22 amino acids and high content of protein. The high profile in phytochemistry with carboxyl, hydroxyl and amino groups serves as an effective reducing agent and capping agent to robust coating on SNPs. Economical- and environmental-friendly approach to synthesize metal nanoparticles terms to be initiative in developing nanotechnology and nanoparticle-based products. In this study, SNPs are biosynthesized in the aqueous extract of *C. vulgaris* using aqueous solution of silver nitrate and characterized for their average particle size, morphology, surface capping, crystal structure and bactericidal properties by UV/Vis spectroscopy, scanning electron microscopy (SEM), transmission electron microscopy (TEM), X-ray diffraction (XRD), Fourier transform infrared spectroscopy (FTIR) and antimicrobial assay.

## Materials and methods

The fresh water green algal strain of *Chlorella* *vulgaris* was collected from Algal Culture Collection, Center for Advanced Studies in Botany, University of Madras, India, and was inoculated in Bold Basal medium. The culture was maintained at 24 ± 1 °C in a thermostatically controlled room and illuminated with cool fluorescence lamps (Phillips 40 W, cool day light 6500 K) at an intensity of 2000 lux in a 16:8 h light/dark regime. In the exponential log phase, when the pigment, protein and carbohydrate measured were maximum, the cells were harvested. The collected cells were washed with double distilled water and sonicated using ultrasonic vibration at 30 % amplitude for 20 min to release the water-soluble biomolecules. The homogenate was subjected to centrifugation (3–4 times), the supernatant was diluted through a series of dilutions with 1 mM AgNO_3_, and the reaction mixtures (10 mL) were incubated at 37 °C.

### Characterization of silver nanoparticles

#### UV–visible spectroscopy analysis

Color changes in reaction mixture were visually observed from white to pale brown then to dark brown. Bioreduction of the silver ions was monitored by sampling 1 mL aliquots at different time intervals (0, 24, 48 and 72 h). The absorption measurements were carried out by Beckman DU 64 UV–visible spectrophotometer.

#### Scanning electron microscopy analysis

Scanning electron microscopic (SEM) analysis of synthesized SNPs was performed using Hitachi S-4500 SEM machine. Thin films of the SNPs were prepared on a carbon-coated copper grid by dropping a very small amount of the aqueous SNPs on the grid, and excess were removed using blotting paper. These films on the SEM grid were then allowed to dry under mercury lamp for 5 min.

#### Transmission electron microscopy analysis

Synthesized SNPs by the biological reduction were prepared for TEM analysis by placing a drop over carbon-coated copper grids and allowing the solvent to evaporate. TEM measurements were performed on a JEOL model 1200EX instrument operated at an accelerating voltage at 80 kV.

#### X-ray diffraction (XRD) measurement

Bio-reduced aqueous silver nitrate solution drop was coated on glass substrate and subjected to XRD measurement using XRD (Model D/Max-2500). Scanning was executed in a 2^θ^ region from 30 to 80º. The pattern was recorded using Cu-K_α_ radiation with a wavelength (λ) of 1.5406 Å at a tube voltage of 40 kV and a tube current of 30 mA. Drop-coated on glass were done on a Phillips PW 1830 instrument operating at a voltage of 40 kV and current of 20 mA with Cu-K_α_ radiation.

#### Fourier transform infrared (FTIR) spectroscopy measurements

After complete reduction in AgNO_3_^−^ ions by aqueous extract of *C.vulgaris*, the reaction mixture was centrifuged at 10,000 rpm for 10 min to isolate the SNPs from free proteins or other compounds present in the solution. The SNPs pellets obtained were freeze-dried and diluted with potassium bromide at 1:100 ratio. FTIR spectrum of samples was recorded on Shimazdu IR Prestige-21 FTIR instrument; all measurements were carried out in the range of 400–4000 cm^−1^.

### Antimicrobial assay

The antimicrobial activity of synthesized SNPs was performed by using agar well diffusion method. About 20 mL of sterile molten Mueller–Hinton agar (HiMedia Laboratories Pvt. Limited, Mumbai, India) was poured into the sterile petriplates. Triplicate plates were swabbed with the overnight culture (10^8^ cells/mL) of human pathogens: *Escherichia coli, Proteus vulgaries, Pseudomonas aeruginosa, Staphylococcus aureus* and *Candida albicans*. The solid medium was gently punctured with cork borer to make a well. Finally, the aqueous SNPs (20 µL) were added into each well and incubated for 24 h at (37° C). After incubation, the zone of inhibition was measured and expressed as millimeter (mm) in diameter.

## Results and discussion

### Synthesis of silver nanoparticles

**Fig. 1:**
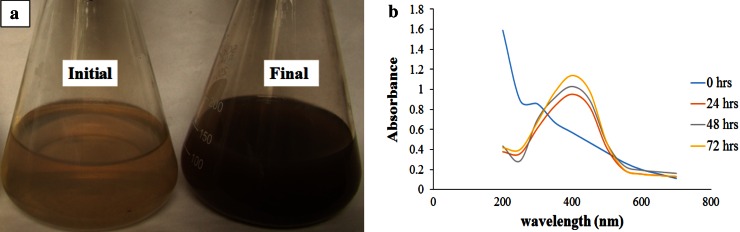
Reaction mixture and UV–visible spectra of the biosynthesized of SNPs

*Chlorella vulgaris* is the most cultivated eukaryotic green microalgae as it is widely used as a health food and feed supplement, as well as in the pharmaceutical and cosmetics industry. It contains proteins, carotenoids, lipid, immune-stimulator compounds, polysaccharides, vitamins, antioxidants and minerals (Mohan et al. 2009). The present study involves the biosynthesis of SNPs where aqueous cell-free extract of *C. vulgaris* was added to the freshly prepared 1 mM silver nitrate (AgNO_3_) at different dilution. The reaction mixture turning brown is a clear indicator for the formation of silver nanoparticles (Fig. 1a) and the reduction in silver ions to silver nanoparticles could be followed by UV–Vis spectroscopy for the confirmation and analysis of nanoparticles (Henglein 1993; Sastry et al. 1998; Ahamed et al. 2003).

### Characterization of silver nanoparticles

UV–visible spectroscopy is one of the most widely used techniques for structural characterization of nanoparticles (Sun et al. 2001). The biosynthesized SNPs were measured by UV–visible spectroscopy at different time intervals to study the change in light absorption and increase in intensity. The absorption spectra of nanoparticles showed highly symmetric single band absorption with peak maximum at 421 nm with steadily increase in intensity as a function of time of reaction without any shift in intensity (Fig. 1b). This indicates the presence of SNPs, which is due to the excitation of surface plasmons (Jae and Beom 2009). After 72 h, no further increase in intensity was recorded, indicating the complete reduction in silver ions.

**Fig. 2:**
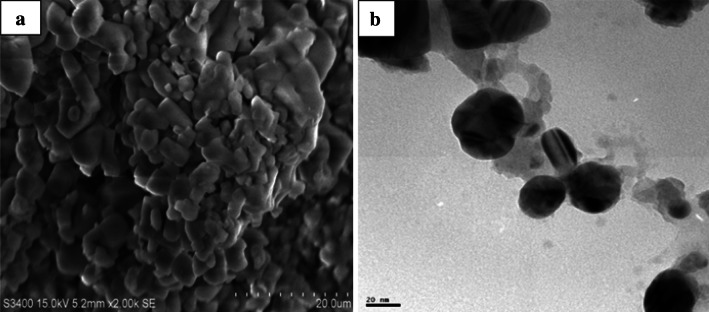
SEM and TEM micrographs of biosynthesized SNPs

Bio-reduced Ag^+^ ions were further characterized for its size, shape, morphology and surface chemistry by SEM and TEM analyses. Surface morphological and nanostructural studies of SEM micrograph showed SNPs to be highly crystalline aggregated spherical SNPs of varied size (Fig. 2a). The large polycrystalline nature of the particles may be due to the fact that on nanometer scale most of the metals are as face-centered cubic (fcc) structures. They tend to nucleate and grow onto twinned and multiply twinned particles with their surfaces bounded by the lowest-energy (111) facets. SNPs have the tendency to agglomerate due to their high surface tension of ultrafine nanoparticles. The fine particle size results in a large surface area that, in turn, enhances the nanoparticle catalytic activity.

**Fig. 3:**
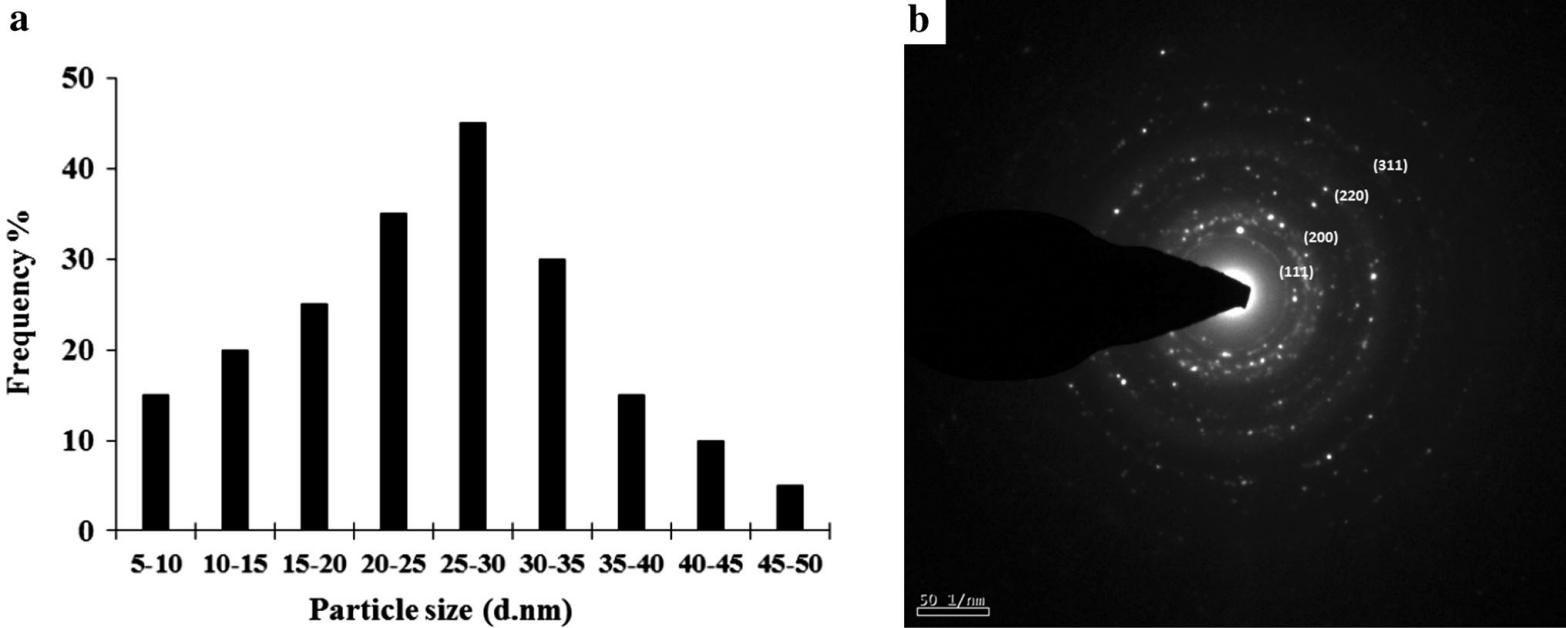
**a** Particle size distribution histogram of SNP’s determined from TEM image **b** SAED showing the characteristic crystal planes of elemental silver

TEM analysis determined distribution, structural features and predominant particle size of SNPs (Fig. 2b). It was noticed that the edges of the particles were lighter than the centers, suggesting that biomolecules such as proteins capped the SNPs. Most of the particles had a size of about 27 nm; the particle size distribution histogram for the SNPs determined from the TEM image is shown in Fig. 3a. From the figure, it is clear that the frequency peak comes at approximately 25–30 nm and particles with sizes ranging from 5 to 50 nm are maximum of the total particles observed. Figure 3b shows selected area electron diffraction pattern (SAED) of the SNPs. The silver particles are crystalline, as can be seen from the selected area diffraction pattern recorded from directing the electron beam perpendicular to one of the nanoparticles in the aggregate. The patterns of SAED spots were indexed according to (111), (200), (220) and (311) reflections of face-centered cubic (fcc) structure of elemental silver on the basis of their d-spacing of 0.232, 0.207, 0.145 and 0.124 nm. The clear and uniform lattice fringes confirmed that the spherical particles are highly crystallized. The lattice spacing of 0.232 nm corresponds to (111) planes of silver and the results reveal the dominant faces of silver spheres are (111). Thus the SNPs synthesized from aqueous algal extract are crystalline spherical particles.

**Fig. 4:**
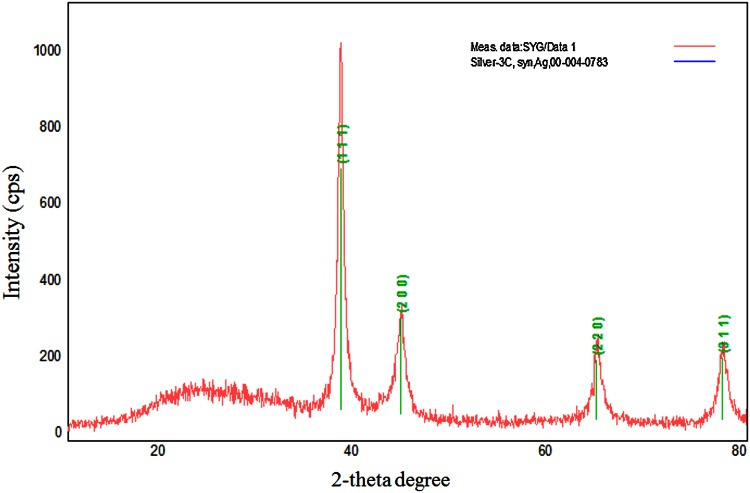
X-ray diffraction of biosynthesized SNPs

The exact nature of the silver particles can be presumed from XRD spectrum of the sample. XRD pattern of the SNPs (Fig. 4) showed four intense peaks in the whole spectrum of 2θ values ranging from 20° to 80°. The peaks of 38.45°, 44.48°, 64.69° and 77.62° correspond to (111), (200), (220), and (311) planes for silver, respectively, illustrating AgNO_3_ complete reduction by aqueous *C. vulgaris* extract to crystalline SNPs (Fig. 3b). Other unassigned peaks could be due to the crystallization of bioorganic phase that occurs on the surface of the nanoparticle. The lattice constant was calculated from the patterns and found to be *a* = 0.4079 nm. This was consistent with the standard value *a* = 0.4086 in agreement with the literature (JCPDS file no. 04-0783). The average particle size was evaluated using the Debye–Scherrer’s formula,

$$D \, = \frac{0.94 \, \lambda }{\beta \, \cos \theta }$$

where *λ* is the wavelength (0.15418 Å) of X-rays used, *β* is broadening of diffraction line measured at half of its maximum intensity (in radian) and *θ* is Bragg’s diffraction angle (in degree) (John and Florence 2009). The crystallite size SNPs are found to be 18.94 nm.

In XRD pattern, the peak breadth is proportional to the mean crystallite size of the material. The sharp and broad areas of the peak typically determine the crystallinity and size of the nanoparticles. The crystallinity of the nanoparticles was evaluated by comparing the crystallite size that was obtained from TEM particle size determination and Debye–Scherrer’s formula.

$${\text{Crystallinity index}}, \, I_{\text{cry}} = \frac{{D_{\text{p}} {\text{ (SEM, TEM)}}}}{{D_{\text{cry}} {\text{ (XRD)}}}} (I_{\text{cry}} \ge 1. 0 0 )$$

where *D*_p_ is the particle size obtained from either TEM or SEM morphological analysis, *D*_cry_ is the particle size calculated from the Deye-Scherrer’s formula (Cullity 1956). If *I*_cry_ value is close to 1, then it is assumed that crystallite size represents monocrystalline, whereas polycrystalline have much larger crystallinity index (Pan et al. 2010). *I*_cry`_ of the biosynthesized SNPs was higher than 1.0, indicating high crystallinity and well-indexed fcc phase structure.

**Fig. 5:**
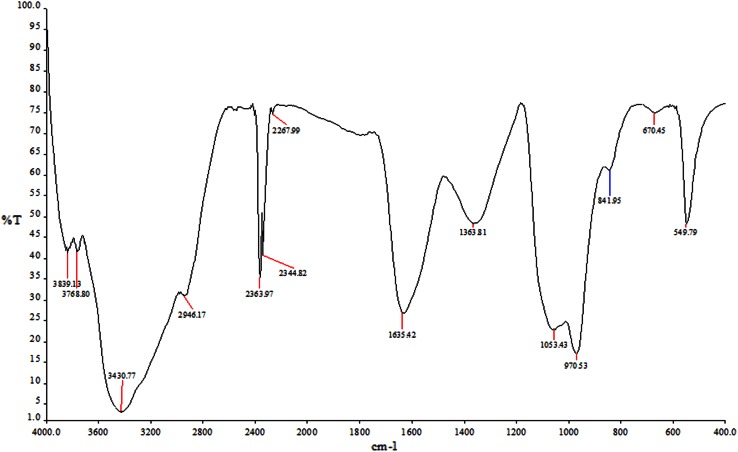
FTIR of biosynthesized SNPs

FTIR measurements were carried out to identify biomolecules responsible for the stabilization of the newly synthesized SNPs. The studies suggest that carbonyl groups of amino acids and peptides have stronger ability to bind metals, and the proteins could form a coat covering metal nanoparticles (Lin et al. 2005). FTIR results of biosynthesized SNPs from aqueous algal extract showed nine prominent bands (Fig. 5) of which broad band at 3430 cm^−1^ corresponds to OH stretching of alcohol or phenol groups, whereas sharp bands at 2363 and 2344 cm^−1^ correspond to C ≡ N, nitriles. Narrow band at 1635 cm^−1^ represents carbonyl N–H stretch of primary amines due to vibrations in amide linkages of protein (Bansal et al. 2005), and 1363 cm^−1^ corresponds to C–H alkane group. The band at 1053 cm^−1^ corresponds to C–N stretching vibrations of aliphatic amines that are commonly found in proteins, indicating the presence of proteins as ligands for SNPs increasing the stability of synthesized nanoparticles, respectively (Huang et al. 2007; Sanghi and Verma 2009). The bands at 970 cm^−1^ attributes to the side-chain vibrations consisting of C–H stretching of alkenes, while bands at 670 and 549 cm^−1^ indicates C–Br stretch of alkyl halides. Bands around 1650, 1550 and 2550 cm^−1^ indicate amide linkages between amino acid residues in protein, and these are well-known signature bands in infrared regions of electromagnetic spectrum. The present finding coincides with the report suggesting that protein can bind to nanoparticles either through free amine groups, cysteine residues or through electrostatic attraction of negatively charged carboxylate groups in the cell-free extracts (Gole et al. 2001). Thus, the release of proteins probably has a role in the formation and stabilization of SNPs in aqueous extract.

### Antimicrobial assay

**Fig. 6:**
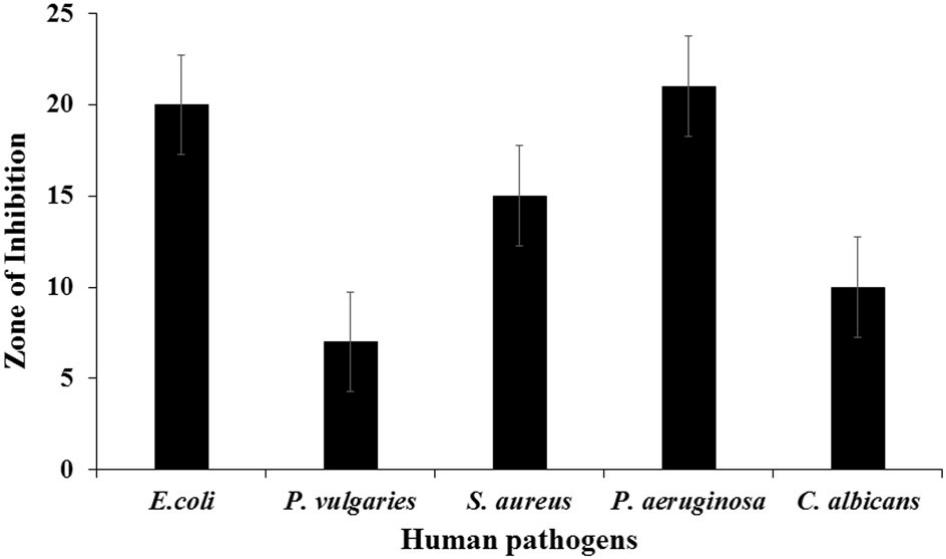
Antimicrobial activity of biosynthesized SNPs

The resistance of human pathogens to the commercially available antimicrobial agents and antibiotics has raised the need to explore new natural and inorganic substitutes to overcome the problem. Among inorganic antimicrobial agents, silver has been widely used to fight against infection since ancient times. Antimicrobial activity of the silver, silver ions, silver compounds has been thoroughly investigated, and surveys have revealed the remarkable antibacterial activity of SNPs. In our investigation, SNPs were found to be toxic to human pathogens (*E. coli, P. vulgaries, S. aureus, P. aeruginosa* and *C. albicans*) and exhibited the maximum inhibition against *P. aeruginosa* of zone 21 mm followed by 20 mm against *Escherichia coli*. Other three human pathogens were moderately susceptible (Fig. 6). These results concurs with the report of Ingle et al. (2008), suggesting that SNPs exhibit significant antimicrobial activity against *E. coli* and multidrug-resistant *S. aureus*. Silver has been also known to exhibit strong toxicity to wide range of microorganisms. SNPs are found to be cytotoxic to *E. coli* and reported to be dependent on size and shape of SNPs (Morones et al. 2005; Pal et al. 2007).

The biosynthesized SNPs from algal aqueous cell-free extract were moderately susceptible to fungal pathogen *C. albicans*, suggesting that the increase in dose may tend to be efficiently toxic. In a study a Kim et al. (2008), SNPs showed potent activity against *Trichophyton mentagrophytes, Trichosporon beigelii* and *C. albicans* which was compared with the commercially available antifungal agents (amphotericin and fluconazole). Stable solutions containing up to 35 ppm SNPs were found to have effective antifungal property against *Aspergillus, Penicillium* and *Trichoderma sps* (Petica et al. 2008). SNPs at 100 ppm totally inhibited the bacterial growth, but the activity against mold and dermatophytes was low; bacteria and molds exhibited resistance against SNPs at 50 ppm concentration (Falkiewicz-Dulik and macura et al. 2008). In case of *C.albicans* also, SNPs are found to be cytotoxic disrupting the cell membrane and formation of pits and pores on membrane surface, subsequently leads to cell death (Kim et al. 2008, 2009). SNPs have found its application against fungal infection and in biostabilization of footwear materials.

## Conclusion

A critical need in the field of nanotechnology is the development of reliable and eco-friendly process for the synthesis of metallic nanoparticles. SNPs are toxic to human beings at high concentration, while nontoxic at low concentration. The growing interest in metallic nanoparticles is due to their exclusive catalytic, optical, electronic, magnetic and antimicrobial properties. In our study, we have investigated the synthesis of silver nanoparticles in a cost-effective approach using extract of *Chlorella vulgaris.* Synthesized SNPs were been characterized to be stable without any impurities and of average size 27 nm. Thus, biosynthesized SNPs can find immense application in the field of biomedical appliances, formulation of antimicrobial agents and in combination with antibiotics.
